# Climate Change and African Agricultural Resilience: A 10‐Year Comprehensive Review of Adaptive Capacity, Policy, and Environmental Stewardship

**DOI:** 10.1002/pei3.70191

**Published:** 2026-07-24

**Authors:** Chibuzor Onyinye Okonkwo, Kahsay Tadesse Mawcha, Rumbidzai Changwa, Dennis Ndolo, Olubukola Oluranti Babalola

**Affiliations:** ^1^ Biopesticides Group International Centre for Genetic Engineering and Biotechnology (ICGEB) Cape Town South Africa; ^2^ Department of Biochemistry, Faculty of Basic Medical Sciences University of Calabar Calabar Nigeria; ^3^ Department of Plant Sciences Aksum University Tigray Ethiopia; ^4^ University of Pretoria Pretoria South Africa; ^5^ Food Security and Safety Focus Area, Faculty of Natural and Agricultural Sciences North‐West University Mmabatho South Africa

**Keywords:** ecosystem stewardship, food security, green technology, indigenous knowledge systems, smart practices, sustainability

## Abstract

In the current era, climate change has emerged as a fundamental threat to African agriculture, driven by increasingly erratic climatic patterns such as prolonged rainfall, decadal droughts, and escalating thermal stress. This review evaluates contemporary agricultural productivity by gathering and analyzing empirical data from academic publications and credible online resources indexed between 2015 and 2025. The primary objective is to delineate the impacts of climatic instability on yield trajectories and food system accessibility. Utilizing a regional comparative framework, the study examines 10 African nations (two per geographic region) to identify localized and pan‐African trends. Data synthesis from Scopus, Web of Science, and Google Scholar reveals that climatic variations have fundamentally undermined food security, contributing to famine, economic displacement, and the erosion of rural livelihoods. Key barriers to effective adaptation were identified as financial incapacity, weak institutional synergy, and a deficit in research and development infrastructure. Consequently, this review highlights the urgent need for a multifaceted resilience strategy that harmonizes indigenous adaptation practices, advanced genomics technology, application of artificial intelligence, and digital climate‐smart technologies. Strengthening food security will require not only technological adoption but also robust policy frameworks, incentivized investment, infrastructural development, and the expansion of extension services to bridge the gap between climate research and on‐farm implementation.

## 1 Introduction

Climatic instability represents a primary constraint on agricultural productivity in Africa, a continent where the sector remains fundamentally dependent on seasonal rainfall. This vulnerability is largely driven by global warming, characterized by rising mean temperatures resulting from the generation and emission of greenhouse gases (GHGs) through human activities. According to the United Nations Environment Programme (2021), adaptation cost for climate change across Africa is estimated to be about 7–15 billion dollars per annum, and 50 billion dollars by 2050 if global warming continues at the current rate. Notably, a significant equity gap exists, as the emissions generated by developing nations are minimal relative to the historical and current contributions of industrialized economies (Mostefaoui et al. 2024). Given the pivotal role of agriculture in economic development and regional employment, addressing these climatic disruptions is essential for continental stability.

Research indicates that an increase in temperature exceeding 1°C could reduce total food output by 3% in developing regions (Baptista et al. 2022; IPCC 2022). Beyond productivity, the nutritional implications are severe, as infants in tropical Sub‐Saharan Africa continue to experience wasting, underweight, and stunted growth as a direct result of climate stressors (Klapka et al. 2026). To achieve the 1.5°C threshold established by the Intergovernmental Panel on Climate Change (IPCC), agricultural stakeholders must synergize to implement best practices that improve adaptive capacity and mitigate climate‐induced migration. Furthermore, systemic socioeconomic issues such as the poor recruitment of youth into the agricultural workforce and high unemployment rate limit household access to food, further entrenching poverty (Masipa 2017). Biologically, climate change facilitates the proliferation and encroachment of agricultural pests into new territories. In Nigeria, for example, crop yields have diminished significantly due to the dual pressures of pest outbreaks and dwindling water bodies caused by protracted drought (Omotoso et al. 2023). In regions characterized by fragile political atmospheres or suboptimal leadership, these environmental pressures often serve as catalysts that contribute to social unrest and political instability (Waha et al. 2017; Scheffran et al. 2019). Consequently, there is an urgent need to develop climate‐smart strategies that align the African agricultural systems with current environmental realities.

While Climate Change (CC) refers to an alteration in weather pattern over a prolonged period, Global Warming deals with the anthropogenic rise in atmospheric temperatures driven mainly by human activities. These two phenomena are addressed by the systemic ability to adjust functions and performance in a way that reduces vulnerability while maintaining productivity. The capacity of agricultural systems to absorb disturbances and recover from hazardous events while maintaining essential functions and structures is referred to as agricultural resilience (FAO et al. 2025).

Climate action in Africa must be woven into the fabric of economic and developmental strategy, as agricultural stakeholders must collaborate to bring actionable, high‐impact policies to the forefront of governance. This study addresses the sustainable development goal number 13, which calls for urgent climate action to combat climate change and its impacts, strengthen resilience to climate hazards, and integrate climate measures into policy as well as improve institutional capacity. The information gathered from this study will be relevant for African governments, policy makers, regulators, researchers, and the general public.

This research aims to evaluate the performance and resilience of the African agricultural sector in the past decade by assessing system exposure and existing adaptive strategies used to develop evidence‐based frameworks for policymakers. Specifically, the study seeks to quantify the vulnerability of agricultural systems to erratic climatic conditions while evaluating current adaptive strategies employed at local, national, and global scales. By identifying the systemic gaps and gray areas that currently impede effective climate adaptation, this research ultimately proposes novel, climate‐smart practices and strategies that could be employed to stabilize agricultural productivity and foster long‐term resilience across the continent.

## 2 Scope and Approach

### 2.1 Study Area

Africa is the world's second‐largest continent, encompassing approximately 30.4 million km^2^ and spanning a significant latitudinal range from roughly 37° N to 35° S. Intersected by both the Equator and the Prime Meridian, the continent's unique geography places a vast majority of its landmass within tropical and subtropical zones, rendering its agricultural systems highly sensitive to climatic fluctuations. The diverse topographical features, ranging from the Mediterranean coast in the north to the confluence of the Atlantic and Indian Oceans in the south, create a complex mosaic of agroecological zones characterized by varying precipitation patterns and thermal regimes (Middleton et al. 2025). To ensure a representative continental analysis, a stratified purposeful sampling approach was used to select two countries across all vulnerability levels from each African region based on their documented vulnerability to climate change.

- West Africa: Ghana and Nigeria.
- East Africa: Ethiopia and Kenya.
- Southern Africa: South Africa and Mozambique.
- North Africa: Egypt and Tunisia.
- Central Africa: Chad and the Democratic Republic of Congo.

### 2.2 Search Strategy, Inclusion and Exclusion Criteria

A comprehensive literature search was carried out to identify relevant studies that capture a multidimensional view of African agricultural resilience, focusing on the intersection of climate science, public policy, and technological innovation. By employing a pairwise country evaluation within each region, the study minimized regional bias and allowed for the validation of intra‐regional trends, while capturing the geographical, climatic, and socio‐economic heterogeneity of the continent. Rather than a systematic protocol, a comprehensive literature search was carried out to identify relevant studies following a thematic search strategy that captures the evolution of the topic. High‐impact review papers were hand‐searched to identify seminal theoretical frameworks not captured in the initial digital search. Details of the search criteria and thematic analysis are summarized in Tables 1 and 2, respectively.

**TABLE 1:** Overview of literature search strategy search terms, and databases used.

Feature	Details
Databases searched	PubMed/MEDLINE, Scopus, Google Scholar, and Web of Science Policy‐specific insights were also captured from the Food and agricultural organization (FAO), World Bank, IPCC, and African Union (AU) databases
Range of search date	January 2015–December 2025
Search terms/keywords	(“Climate Change” OR “Climatic Variability” OR “Global Warming”) AND (“Agriculture” OR “Food Systems” OR “Crop Production”) AND (“Africa” OR “Sub‐Sahara Africa”) AND (“Resilience” OR “Adaptation” OR “Adaptive Capacity” OR “Mitigation” OR “Climate‐Smart Agriculture” OR “Sustainability” OR “Environmental Stewardship” OR “Food Security” OR “Agricultural Policy” OR “Crop Breeding” OR “Plant Breeding” OR “Genomics” OR “Precision Agriculture” OR “Artificial Intelligence”)
Language	English language only
Inclusion criteria	Peer‐reviewed journal articles, meta‐analyses, and official policy reports from international organizations (FAO, WHO, IPCC, World Bank, European Union) that specifically focused on: the African continent, impact of climate change on agricultural productivity, resilience strategies, climate‐smart technologies, and policy evaluations. Studies providing empirical evidence, longitudinal data, or robust theoretical frameworks. Research discussing socio‐economic factors like youth migration, gendered impacts, and extension services
Exclusion criteria	Non‐peer‐reviewed blog posts, opinion pieces, and conference abstracts without full text data. Studies focused on non‐African regions. Studies with unclear methodologies or those lacking a substantive discussion on climate stressors. Purely meteorological studies without an agricultural link or field‐level application. Papers focusing solely on industrial large‐scale farming with no relevance to African smallholder systems
Filter	Duplicate entries were removed during the initial screening phase, and the selected literature was synthesized to identify systemic gaps in adaptive capacity and to propose evidence‐based strategies for enhancing agricultural resilience across the continent

*Note:* This table summarizes the literature search strategy used for the review, including; search terms, databases and key words.

**TABLE 2:** Summary of thematic analysis and supporting references.

Major theme	Subtheme	Key findings	Challenges	Future directions	References
Climate change impacts on African agriculture	Crop productivity and food security	Climate change has reduced agricultural productivity through drought, floods, heat stress, erratic rainfall, and pest outbreaks, threatening food security across Africa	Climate variability, dependence on rain‐fed agriculture, vulnerable farming systems	Develop climate‐resilient production systems and improve adaptation planning	Ahmed et al. (2024); Omotoso et al. (2023); Onyeaka et al. (2024); Ani et al. (2022); FAO et al. (2025)
	Water scarcity	Water shortages significantly reduce crop productivity	Poor irrigation infrastructure and increasing drought frequency	Promote efficient irrigation technologies and water conservation	Brempong et al. (2023); Collins et al. (2024); Mohamed et al. (2025)
	Soil degradation	Climate change accelerates erosion, nutrient depletion, and land degradation	Unsustainable farming practices	Soil restoration and conservation agriculture	Byaro et al. (2023); Mostefaoui et al. (2024); Abdela et al. (2024)
Climate adaptation strategies	Climate‐smart agriculture	CSA enhances resilience while maintaining productivity and reducing greenhouse gas emissions	Low adoption, limited extension services	Scale‐up CSA adoption through policy support and farmer education	FAO (2015); CIAT and World Bank (2017); Mensah et al. (2025); Gebre et al. (2023)
	Improved crop varieties	Stress‐tolerant cultivars improve resilience and food production	Slow breeding programs and seed accessibility	Accelerate breeding and seed dissemination	Raza et al. (2025); Olanrewaju et al. (2022); Wang et al. (2024)
	Water‐smart agriculture	Improved irrigation and water harvesting increase resilience	Infrastructure costs and water availability	Invest in sustainable irrigation technologies	Brempong et al. (2023); Collins et al. (2024)
Indigenous knowledge systems	Indigenous climate adaptation	Indigenous knowledge complements scientific adaptation strategies	Poor documentation and limited policy integration	Integrate indigenous and scientific knowledge systems	Apraku et al. (2021); Ijatuyi et al. (2025); Phakathi and Sinyolo (2025)
	Community resilience	Traditional farming systems improve adaptive capacity	Loss of indigenous knowledge among younger generations	Strengthen community‐based adaptation programs	Balasha et al. (2023); Luu et al. (2024)
Governance, policy and climate finance	Climate finance	Financial investment supports resilient agricultural development	Poor governance, inadequate funding, weak accountability	Improve financing mechanisms and monitoring frameworks	Mungai et al. (2022); Masio (2023); IMF (2024)
	Policy implementation	Effective policies strengthen adaptation capacity	Weak institutions and fragmented governance	Improve evidence‐based policy implementation	Adom et al. (2025); Bello (2025); Omokaro (2025)
	Stakeholder collaboration	Multi‐sector partnerships improve climate resilience	Institutional fragmentation	Strengthen public‐private‐academic partnerships	Luu et al. (2024); Kariuki et al. (2023)
Youth and rural development	Youth engagement	Youth participation promotes agricultural innovation and sustainability	Rural‐urban migration and limited employment	Develop rural infrastructure and agribusiness opportunities	FAO (2024); Mensah et al. (2025)
	Extension services	Knowledge transfer improves technology adoption	Weak agricultural extension systems	Modernize extension services using digital technologies	FAO (2015); Mrisho et al. (2020)
Soil health and plant–microbe interactions	Beneficial microbes	Plant‐growth‐promoting microorganisms enhance stress tolerance and productivity	Limited commercialization and field validation	Expand microbial biotechnology research	Fadiji et al. (2022); Olarenwaju et al. (2024)
	Sustainable soil management	Healthy soils improve resilience and sustainability	Soil degradation and excessive agrochemical use	Promote regenerative agriculture	Brempong et al. (2023); Abdela et al. (2024)
Artificial intelligence and digital agriculture	AI‐assisted breeding	AI improves genomic prediction, breeding accuracy, and crop selection	Limited digital infrastructure and technical capacity	Expand AI‐assisted breeding systems	Fu et al. (2025); Feng et al. (2024); Kaushik (2025)
	Precision agriculture	AI enables disease diagnosis, remote sensing, and precision farming	Technology affordability and digital literacy	Increase access to digital agricultural technologies	Abdul‐Gafaar et al. (2025); Gamage et al. (2024); Mrisho et al. (2020)
	Robotics and automation	Robotics accelerate breeding efficiency and reduce labor requirements	High implementation costs	Integrate robotics into breeding pipelines	Fu et al. (2025); Gamage et al. (2024)
Advanced plant breeding technologies	Genome editing	CRISPR and gene editing accelerate development of climate‐smart crops	Regulatory and biosafety concerns	Harmonize regulations and increase investment	Raza et al. (2025); Wang et al. (2024)
	Multi‐omics technologies	Genomics, transcriptomics, metabolomics, and phenomics improve trait discovery	High analytical costs	Integrate multi‐omics with AI‐assisted breeding	Raza et al. (2025); Fu et al. (2025)
	High‐throughput phenotyping	Automated phenotyping improves breeding precision	Infrastructure limitations	Expand phenotyping facilities in Africa	Wang et al. (2024); Fu et al. (2025)
Sustainable agricultural systems	Renewable energy	Renewable energy improves sustainability and reduces emissions	Poor infrastructure and financing	Promote renewable energy adoption in agriculture	Chaa Kyire et al. (2025); FAO (2026); Mungai et al. (2022)
	Greenhouse gas mitigation	Sustainable farming reduces agricultural emissions	Limited implementation of mitigation strategies	Encourage climate‐smart mitigation practices	Abdela et al. (2024); Pophiwa et al. (2025); Jones et al. (2023)
Future directions and research priorities	Technology convergence	AI, robotics, genomics, synthetic biology, remote sensing, and microbial engineering are converging to revolutionize crop improvement	Fragmented implementation and unequal technology access	Develop integrated digital breeding ecosystems	Fu et al. (2025); Raza et al. (2025); Gamage et al. (2024); Wang et al. (2024)
	Monitoring and evaluation	Strong monitoring systems ensure effective implementation of climate programs	Poor accountability and inefficient resource allocation	Develop standardized monitoring and evaluation frameworks	Luu et al. (2024); Omokaro (2025)
	Knowledge gaps	Limited empirical data from several African regions, particularly Somalia and North Africa, and insufficient farmer‐level evidence	Regional research imbalance	Conduct participatory and field‐based research involving farmers and stakeholders	Mensah et al. (2025); Gebre et al. (2023); Ijatuyi et al. (2025)

## 3 Vulnerability Trends in African Agriculture Between 2015 and 2025

**FIGURE 1:**
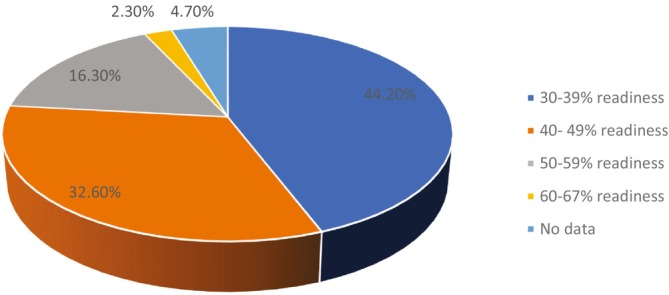
Representation of climate change readiness across African Nations (created from data obtained from section 3.0 above by Birch 2024). This figure shows the level of readiness of African countries toward Climate Change adaptation. Most African countries (about 76.8%) possess a readiness index below 50.0%. This reveals the need for more concerted efforts toward Climate change mitigation by all agricultural stakeholders in Africa.

The impact of climatic instability on African agriculture is profound, directly undermining a sector critical for economic empowerment, food security, and rural livelihoods. Africa's vulnerability to climate stressors is exacerbated by prevailing sociopolitical and economic constraints that amplify the severity of environmental shocks, thereby entrenching poverty and food insecurity (Sultan and Gaetani 2016). A comprehensive assessment by Birch (2024), underscored a significant lack of institutional preparedness across the continent, revealing that most African nations are ill‐equipped to manage the intensifying hazards associated with climatic variability. Nineteen of the 43 countries surveyed, including, Kenya, Chad, and Sierra Leone were categorized as highly vulnerable, with readiness scores ranging between 30% and 39%. A secondary tier, exhibiting marginally higher but still insufficient preparedness (40%–49%), included regional anchors like Nigeria, Ethiopia, and Mozambique (Figure 1). More robust institutional frameworks were observed in nations such as Algeria, Egypt, and Tunisia among others, demonstrating readiness scores between 50% and 59%. Notably, Botswana emerged as the most prepared nation in the assessment, with a readiness index of 60%–69%. Conversely, a critical data deficit was noted for Somalia and Western Sahara, highlighting the challenges of monitoring climate vulnerability in conflict‐affected or data‐poor regions. These disparities in adaptive capacity emphasize the urgent need for targeted, region‐specific interventions to bolster continental resilience (Birch 2024).

Approximately 76.8% of African nations possess a readiness index below 50.0%. This underscores a substantive disparity in global climate resilience, particularly when contrasted with European nations, most of which maintain preparedness levels exceeding 70.0% despite higher historical contributions to global anthropogenic emissions (Jones et al. 2023). To align with the mandates of the Paris Climate Agreement, and ensure food security and a sustainable agricultural system, adaptation efforts within the African context must be critically accelerated and prioritized (Kariuki et al. 2023).

### 3.1 Climate Change Vulnerability Status in Selected African Nations

#### 3.1.1 Ethiopia

In Ethiopia, climate change is perceived as a dualistic phenomenon: a systemic threat to an agro‐dependent economy and a marginal opportunity due to altered precipitation patterns that occasionally extend planting and harvesting windows (Alemu and Mengistu 2019). However, the negative impacts predominate. Southern Ethiopia has faced severe threats to household food security over the last three decades, with significant yield contractions in staple crops such as sorghum and barley (Mekonnen et al. 2021). The Malthusian pressure of a geometrically increasing population, coupled with resource scarcity, necessitates robust mechanisms to absorb climatic shocks (Ahmed et al. 2024). Over 70% of Ethiopia's population depends on rain‐fed agriculture, and climate vulnerability is tightly linked to immediate food and economic security. Currently, erratic weather has reduced rainfall in Ethiopia by 15%–20% during critical planting cycles (Mekonnen et al. 2021), while extreme events like El Niño continue to exacerbate the frequency and severity of droughts in the country (Gitima and Mersha 2020).

#### 3.1.2 Egypt

Egypt's agricultural sector faces severe structural setbacks without intensified adaptation efforts. In a recent study by Abou‐Hadid (2025), the author highlighted the deleterious impact of long drought seasons, varying temperatures, and rising sea levels on Egyptian agriculture (Abou‐Hadid 2025). These impacts are disproportionately felt by lower‐income cohorts who lack the fiscal capacity to mitigate environmental risks (Hamzawy et al. 2023). Characterized by an arid desert and dwindling groundwater reserves, Egypt is currently navigating a loss of arable land driven by rapid urbanization and population pressure. This has compromised national self‐sufficiency, leading to an over‐reliance on food imports and increased exposure to global price volatility (Salah et al. 2025). A resilient future for Egyptian agriculture requires an integrated framework that harmonizes environmental policy with technological and financial reforms in addition to climate‐smart agricultural practices.

#### 3.1.3 Tunisia

Tunisia is categorized among the most water‐stressed nations globally, a condition exacerbated by escalating mean temperatures and anthropogenic climate change (Sakir et al. 2025). Between 2013 and 2017, the nation experienced protracted droughts, intensified heatwaves, and wildfires, which severely jeopardized agricultural output (Ouessar et al. 2021). Because the sector relies heavily on rain‐fed systems, it remains hypersensitive to climatic fluctuations. Furthermore, rising sea levels have induced saltwater intrusion into coastal aquifers, leading to harmful soil salinization and long‐term ecosystem degradation (Mohamed et al. 2025).

#### 3.1.4 Chad

Chad's economy is fundamentally agro‐dependent, with the sector employing 75% of the workforce and contributing 40% to the national gross domestic product (GDP). Paradoxically, while Chad contributes only 0.2% to global greenhouse gas emissions, it remains one of the world's most vulnerable nations to climatic shifts (International Monetary Fund 2024). This vulnerability is driven by a combination of high thermal stress, low adaptive capacity, and increasing precipitation irregularity (Cooper and Price 2019). Recent trends indicate rapid soil degradation and natural habitat loss, which have precipitated communal conflicts over dwindling food and water resources (Byaro et al. 2023).

#### 3.1.5 Democratic Republic of Congo (DRC)

Data from 2015 to the present indicate significant shifts in the DRC's precipitation pattern (Balasha et al. 2023). In 2019, delayed and unevenly distributed rainfall resulted in widespread yield failures and increased pest infestations, compromising crop quality (Chadwick et al. 2016). These disruptions have a distinct gendered impact, as women, who disproportionately depend on marshland agriculture for economic autonomy, face heightened financial vulnerability due to these precipitation anomalies (Adom et al. 2025).

#### 3.1.6 Ghana

Ghana's agricultural sector is characterized by low mechanization and a critical infrastructure deficit, with < 5% of farmland currently under irrigation (Abdul‐Gafaar et al. 2025). This structural fragility renders the sector highly susceptible to erosion and land degradation (Mensah et al. 2025). Climate Change has also induced food price inflation, driven in part by the rising energy costs associated with mitigation efforts (Sarku 2023). The adoption of climate‐smart techniques such as; drought‐resistant cultivars and improved hydrological management‐ is currently hampered by significant funding gaps (Owusu et al. 2024; Chaa Kyire et al. 2025). However, smallholder farmers have already begun adopting adaptive measures within their financial capacity, including the cultivation of improved crop cultivars, dry‐season farming, mixed cropping, and the utilization of social networks for knowledge exchange (Owusu et al. 2024).

#### 3.1.7 Nigeria

In Nigeria, climate stressors have manifested as reduced crop yields, increased pest prevalence, and severe land degradation. The northern region, which is the nation's primary agricultural hub, is particularly affected by altered precipitation and protracted drought (Omokaro 2025). These conditions have served as catalysts, propelling social unrest, malnutrition, and broader national insecurity (Ani et al. 2022). Specifically, the loss of green vegetation has disrupted traditional transhumance patterns, forcing herders into the southern regions and escalating communal conflicts, which further destabilizes the national food system (Bello 2025).

#### 3.1.8 South Africa

Compared to their regional peers, South Africa exhibits higher adaptive capacity and by extension greater resilience relative to many other African countries due to the institutional prioritization of food security in the country. However, shifts in precipitation and thermal regimes are already reducing crop suitability in western and central production belts (Trisos et al. 2022). Projections suggest that staples like maize, as well as high‐value viticulture and horticulture zones, will face continued yield contractions (Zenda 2024). Smallholder farmers remain the most vulnerable demographic, facing significant barriers to survival as climate stress undermines traditional agricultural livelihoods (Zenda and Rudolph 2024).

#### 3.1.9 Mozambique

The 2019 landfall of Cyclone Idai serves as a benchmark for climate‐induced catastrophe in Mozambique, resulting in the displacement of over 1 million citizens and the total destruction of unharvested crops and livestock (Sunu et al. 2025). The nation's reliance on rain‐fed agriculture exposes its agricultural GDP to significant volatility (Detelinova et al. 2023; CIAT, World Bank 2017). Prolonged harvest failures have turned previously fertile regions into arid landscapes, forcing an increased reliance on international humanitarian assistance and undermining the socio‐economic fabric of rural communities (FAO 2024).

#### 3.1.10 Kenya

**FIGURE 2:**
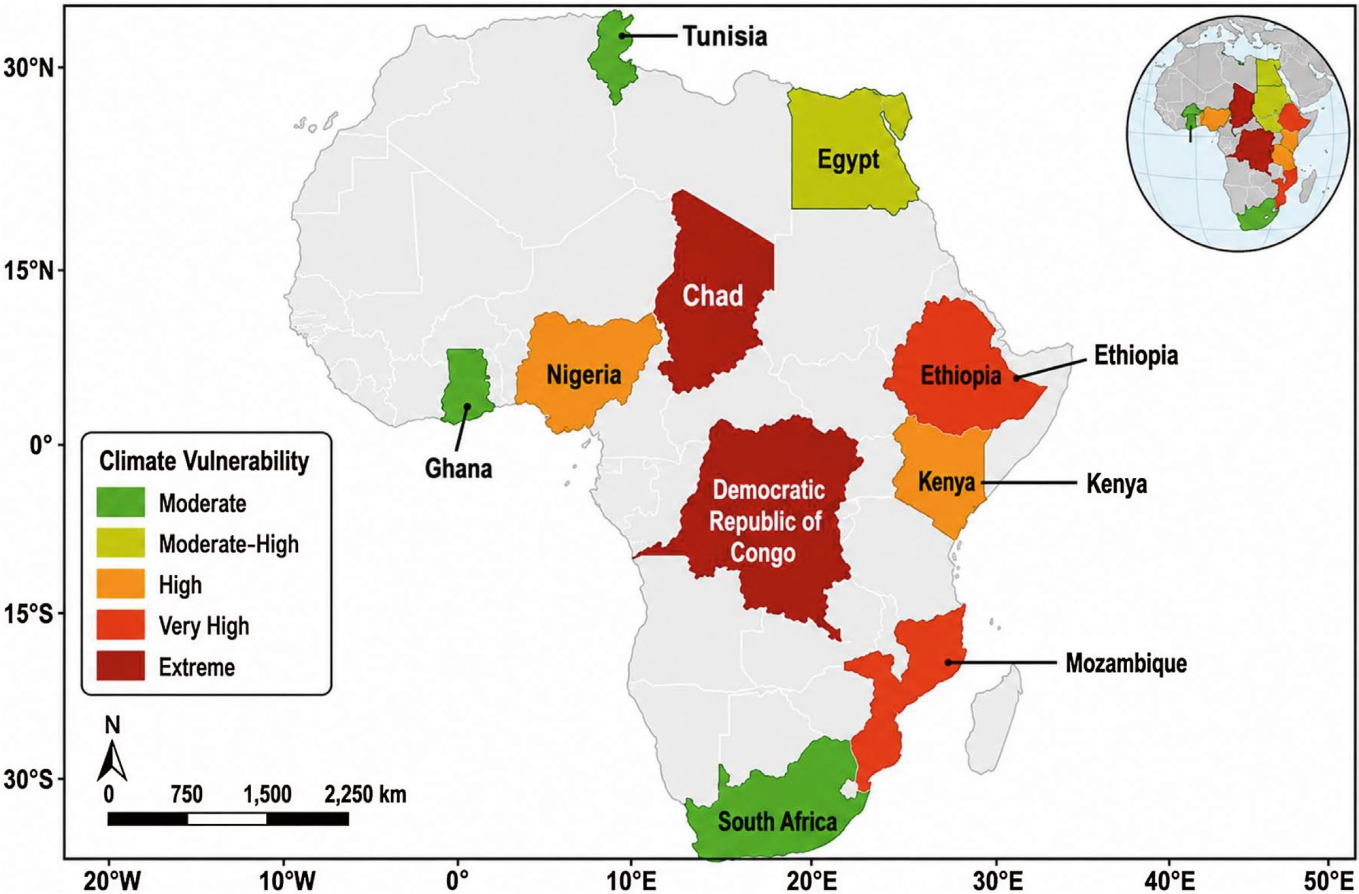
Climate change vulnerability status of representative African counties. (Designed by authors using ChatGPT). This figure shows climate vulnerability trends across studied African countries. While a few countries like South Africa, Tunisia, and Ghana are moderately vulnerable to climate change effects, others such as Chad, DRC, Ethiopia, and Mozambique need more urgent actions/interventions to build stronger adaptive, mitigative, and resilient structures that reduce their vulnerability status and secure food for their nations.

Kenya has experienced a significant cryospheric loss, with Mount Kenya's glaciers retreating by approximately 90% in recent decades. Current glaciological records indicate a persistent retreat that may lead to complete deglaciation in the near term (Prinz et al. 2018). Climate Change has threatened Kenya's food production and sustainable agriculture as Kenyan farmers depend on rain‐fed agriculture for survival. Some of the adaptive strategies employed by Kenyan farmers to fight climate change impact include planting drought‐tolerant crop varieties, use of diversified crops, growing early maturing crops, and diversifying sources of livelihood (Gebre et al. 2023). These strategies are however mostly employed by young and educationally enlightened farmers, leaving the more elderly and less enlightened farmers to their fate (Gebre et al. 2023). This reiterates the need for agricultural policies that increase farmers' awareness and give them access to education and agricultural extension services. The climate vulnerability map for representative African countries is shown in Figure 2. A critical comparison of regional trends, identifying common barriers and unique success factors across the studied geographies is summarized in Table 3.

**TABLE 3:** Critical comparison of regional agricultural trends & resilience in Africa.

Region	Studied countries	Key vulnerabilities & trends	Common barriers (cross‐regional)	Unique success factors & adaptive strategies
West Africa	Ghana, Nigeria	High prevalence of crop pests and land degradation. Protracted drought in Northern Nigeria and disrupted transhumance patterns. Low mechanization and poor irrigation (< 5%) in Ghana	Fiscal constraints including high debt‐to‐gross domestic product (GDP) ratios limiting climate investment. Institutional siloing, and poor coordination between research and policy Infrastructure deficit including the lack of storage and transport networks	Digital innovation pioneering the use of AI platforms like *Hello Tractor* for mechanization The use of social networks for farmer‐to‐farmer knowledge exchange
East Africa	Ethiopia, Kenya	Significant cryosphere loss (90% retreat of mount Kenya's glaciers). Erratic weather causing 15%–20% reduction in rainfall during planting cycles. Malthusian pressure from rapid population growth	Youth migration from rural to urban settlements is depleting the agricultural labor force. Information gaps, exacerbated by lack of access to extension services for elderly/uneducated farmers	High adoption of drought‐tolerant and early‐maturing crop varieties Technology adoption including high diagnostic accuracy using AI apps like *Plant Village Nuru*
Southern Africa	South Africa, Mozambique	Extreme events such as the Cyclone Idai (Mozambique) as a benchmark for climate catastrophe. Shifting suitability for staples (maize) and high‐value viticulture	Funding gaps and slow disbursement of international climate funds Data deficits and challenges in monitoring vulnerability in data‐poor areas	Successful harmonization of indigenous knowledge systems (IKS) with modern irrigation Institutional priority and high adaptive capacity due to national prioritization of food security
North Africa	Egypt, Tunisia	Extreme water scarcity and saltwater intrusion into coastal aquifers. Rapid urbanization leading to the loss of fertile arable land	Exposure to global price volatility due to compromised self‐sufficiency and import dependency Poor funding of Research and Development (R&D) as well as infrastructural deficit for advanced climate modeling	Robust frameworks with the highest regional readiness scores (50%–59%) in institutional preparedness. Integrated policy that harmonizes environmental policy with financial reforms
Central Africa	Chad, DRC	High sensitivity as economies depends fundamentally on agriculture (75% of workforce). Delayed/uneven rainfall leading to widespread yield failures	Fragile leadership including environmental pressures serving as catalysts for social unrest and conflict. Gendered impact, with women disproportionately affected by precipitation anomalies	Agroecology advocacy including; transitioning to biopesticides and organic soil amendments Carbon Sequestration with focus on agroforestry and forest conservation

### 3.2 Current Adaptation/Mitigation Strategies to Build Stronger Agricultural Resilience

To mitigate the deleterious impacts of climate instability on agriculture in Africa, the following multidimensional strategies have been employed.

#### 3.2.1 Transition to Renewable Energy Systems, Circular Economy and Waste Management

Decarbonizing the agricultural value chain through the integration of solar, wind, and geothermal energy offers significant environmental and socio‐economic benefits. Replacing fossil‐fuel‐dependent power generation with renewable alternatives directly reduces the atmospheric accumulation of GHGs. This necessitates a strategic shift toward energy‐efficient infrastructure and electric mobility, which collectively preserve local biodiversity and enhance rural health outcomes. FAO has advocated the integration of renewable energy into agricultural systems to enhance environmental sustainability, food security, and economic development. This has enabled beneficiary countries to increase productivity and reduce emissions (FAO 2026). Circular agricultural models, such as the recycling of food waste into bio‐fertilizers, mitigate methane emissions while enhancing soil sequestration. Additionally, nature‐based solutions such as using organic fibers for erosion control offer sustainable methods for ecosystem preservation without the negative externalities associated with synthetic agrochemicals (Jackson 2025).

#### 3.2.2 Multilateral Policy Frameworks and Digital Technological Approach

Effective climate action requires a tripartite approach: aggressive GHG mitigation, the adoption of Climate Smart Agriculture (CSA), and robust resource mobilization. Global monitoring indices are essential for tracking regional progress and ensuring the timely dissemination of meteorological warnings (Alemu and Mengistu 2019). Predictive meteorological modeling serves as an anticipatory governance tool that allows farmers to align planting cycles with shifting precipitation patterns. Early warning systems (EWS) facilitate risk‐informed decision‐making, thereby minimizing crop loss and maximizing resource efficiency (Collins et al. 2024). Furthermore, high‐emitting industrialized nations bear a disproportionate responsibility to provide climate finance and technical support to developing regions, fostering an integrated management approach that prioritizes innovative research and development as well as policy reforms (Alemu and Mengistu 2019). This includes the adoption of digital meteorological advisories, precision water management (e.g., rainwater harvesting and rerouting infrastructure), and time‐efficient mechanical tools (Abdela et al. 2024; Nji et al. 2022).

#### 3.2.3 Adoption of Agroecological, Environmental, and Climate‐Smart Techniques

FAO advocates for a synergy between mitigation efforts and food security frameworks. This includes transitioning from chemical‐intensive agriculture to agroecological practices, such as the use of biopesticides and organic soil amendments. The use of compost manure has been reported to improve soil fertility, maintain diversity of microbes and biomass activity relative to their synthetic counterparts (Raza et al. 2025). These methods reduce the carbon footprint of the sector while preserving soil and water quality. Forest conservation and afforestation are vital for carbon sequestration, serving as natural heat sinks that regulate local precipitation patterns. Agroforestry, which integrates perennial woody plants into crop and livestock systems, is estimated to remove substantial volumes of CO_2_ from the atmosphere by mid‐century (Abdela et al. 2024). Furthermore, the application of microbial biostimulants offers a potential mechanism to protect crops from climate‐induced abiotic stress while reducing reliance on conventional pesticides (Fadiji et al. 2022). Also, because of the symbiotic relationship between legumes and rhizobia, which provides a renewable source of nitrogen to the soil, including legumes in crop diversification improves soil fertility and quality (Raza et al. 2025), thus improving nutrient cycling, pest suppression and water‐use efficiency.

#### 3.2.4 Research, Knowledge Transfer and Human Capacity Building

Strengthening resilience at the local level requires a concerted effort to enhance human capital through education and extension services (Ani et al. 2022). Initiatives like the Communication for Development (ComDev) tool in the DRC, alongside Farmer Field Schools, bridge the informational gap between researchers and smallholders, ensuring that climate‐smart innovations are effectively disseminated and adopted (FAO 2015). Human capacity building via research and development (R&D) is a primary driver of agricultural transformation. Molecular biology and genomic sequencing offer profound potential for unlocking the genetic resilience of “orphan crops” such as the Bambara groundnut, enabling the development of highly drought‐tolerant cultivars (Olarenwaju et al. 2024). Furthermore, rhizosphere engineering, the manipulation of plant root metabolomes and microbiota, presents an eco‐friendly pathway to enhance crop adaptation under abiotic stress (Olanrewaju et al. 2022).

### 3.3 Systemic Obstacles to Climate Adaptation/Mitigation in Africa

Despite the availability of adaptive frameworks and mitigation options to Climate Change impact in Africa, several structural impediments hinder the effective operationalization of climate resilience in Africa. Some of these obstacles include:

#### 3.3.1 Fiscal Constraints and the Adaptation Finance Gap

The ability of African nations to invest in climate resilience is severely compromised by fiscal limitations and global economic volatility. The global downturn, food crisis, and poor government policies are some of the factors that have impeded the ability of the continent to counteract climate change effects so far (Woodhill et al. 2022). It is estimated that coastal African nations require between 5% and 10% of their GDP to effectively mitigate climate‐driven risks. However, the slow disbursement of international climate funds and the lack of full commitment from high‐emitting developed nations remain significant barriers (Mungai et al. 2022).

#### 3.3.2 Institutional Fragmentation and Stakeholder Siloing

**FIGURE 3:**
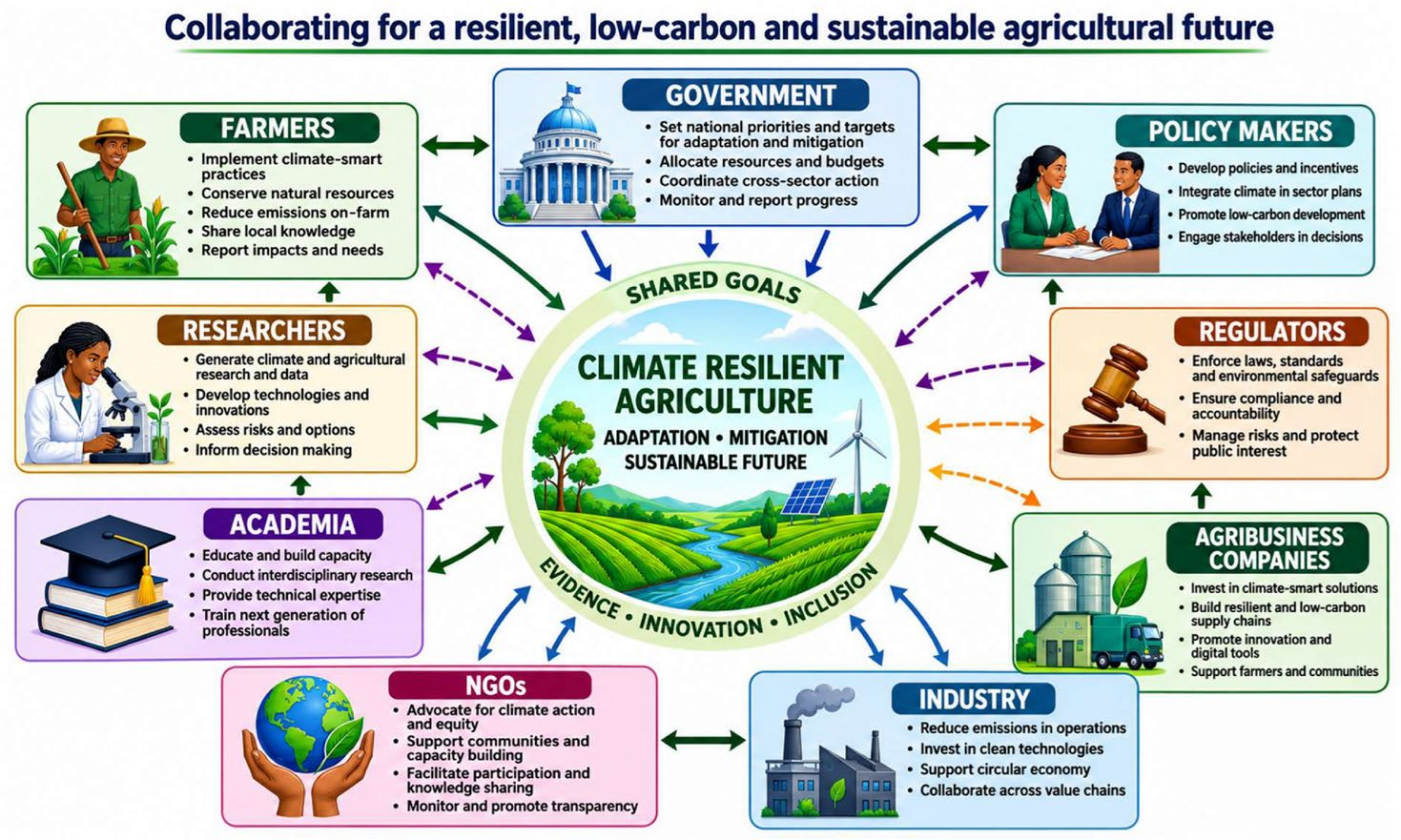
Illustrative diagram showing key stakeholders and their roles in climate change adaptation and mitigation (Designed by authors using ChatGPT). This figure illustrates the key stakeholders whose coordinated efforts are essential for building a more resilient, low‐carbon, and sustainable agricultural future in Africa. The figure also highlights the complementary roles and interrelationships of each stakeholder in driving climate change mitigation and adaptation, promoting agricultural innovation, strengthening food systems, and enhancing food and nutrition security. Through effective collaboration and coordinated action, these stakeholders can accelerate the transition toward a productive, climate‐resilient, and sustainable agricultural sector across the continent.

Effective climate management requires transdisciplinary collaboration (Figure 3). Currently, adaptation efforts are often undermined by fragmented coordination between government bodies, research institutions, academia, and local practitioners (Luu et al. 2024). This “institutional siloing” prevents the integration of scientific research into actionable policy, complicating the management of regional GHG emissions and causing disruptions in agricultural activities, especially in the African region (Pophiwa et al. 2025).

#### 3.3.3 Deficits in Research Infrastructure and Intellectual Capital

In many African nations, R&D remains underfunded, leading to a deficit in the scientific infrastructure required for climate modeling and bio‐innovation. In countries like Nigeria, chronic underfunding of tertiary institutions which are the major research hubs has resulted in massive migration of intellectuals to more developed nations, as specialized researchers seek better‐equipped facilities abroad. Addressing this requires an urgent prioritization of scientific investment to build the human capacity necessary for long‐term agricultural resilience in Africa (Onyeaka et al. 2024).

#### 3.3.4 Limited Adoption of Advanced Precision and Genomics Technologies

While many developed countries have adopted advanced crop breeding strategies based on genomic selection, marker‐assisted breeding, and multi‐omics integration, much of Africa and other developing regions continue to rely predominantly on conventional breeding approaches that emphasize empirical phenotypic selection (Fu et al. 2025). This technological gap limits the efficient application of precision‐driven breeding, as the development and interpretation of deep learning models require high‐quality datasets, robust computational infrastructure, and interdisciplinary expertise, all of which remain insufficient in many African research institutions. Furthermore, generating accurate predictions within high‐dimensional genomic datasets, where the number of genetic markers greatly exceeds the breeding population size, remains a major technical challenge (Feng et al. 2024).

Despite these constraints, advanced genomics technologies have the potential to revolutionize crop improvement by accelerating the development of high‐yielding, climate‐resilient, and nutritionally enhanced crop varieties capable of meeting future food security demands (Feng et al. 2024). Realizing this potential, however, requires substantial initial investment in infrastructure, high‐throughput phenotyping platforms, multimodal AI model development, computational resources, and the calibration of automated robotic breeding systems. Such foundational investments are essential for establishing scalable, gene‐enabled breeding pipelines capable of delivering sustained improvements in breeding efficiency and crop innovation (Fu et al. 2025).

#### 3.3.5 Inadequate Adoption and Explainability of AI Technologies

Despite the transformative potential of artificial intelligence (AI) in modern crop breeding, its adoption remains constrained by concerns regarding the transparency, interpretability, and reliability of AI‐driven decision‐making. Although AI offers exceptional capabilities for processing large‐scale datasets and optimizing breeding decisions, its outputs must be understandable, traceable, and scientifically justifiable, particularly in applications with significant ethical, environmental, and societal implications (Fu et al. 2025). Consequently, the development and adoption of explainable AI are essential for enabling breeders and researchers to understand the rationale underlying AI‐generated recommendations, thereby improving confidence, accountability, and trust in AI‐assisted breeding systems. Future AI models should integrate global expertise, biological knowledge, and empirical evidence while ensuring transparent, interpretable, and controllable decision‐making processes. Such knowledge‐driven AI systems will empower breeders to address complex challenges at the interface of genetics, ecology, climate science, and ethics through evidence‐based decision‐making, ultimately reducing uncertainty, mitigating unintended consequences, and promoting the responsible deployment of AI in climate‐smart crop improvement (Fu et al. 2025).

## 4 Future Perspectives for African Agriculture

Evidence from this study elucidates the vulnerabilities and emerging opportunities defining Africa's agricultural trajectory amidst a changing climate (Table 3). While certain regions, such as Ethiopia and parts of Southern Africa, may benefit from extended growing seasons due to shifting climatic patterns, these localized gains are largely eclipsed by widespread threats to food production, rural livelihoods, and environmental stability (Trisos et al. 2022). Consequently, the continent requires comprehensive adaptation strategies underpinned by innovation, robust institutional frameworks, integrated knowledge systems (Oniang'o et al. 2025), and intensive human capacity development for farmers and other sector players. Deficiencies in irrigation networks, storage facilities, and transport systems restrict the ability of farmers to respond effectively to environmental shifts. Furthermore, low sovereign credit ratings often hinder the flow of international climate finance into the agricultural sector. A primary theme emerging from this analysis is the necessity of harmonizing indigenous knowledge with contemporary technological advancements. A common finding in the Southern region, especially South Africa, is that farmers who integrate traditional practices with modern agricultural systems achieve superior outcomes compared to those utilizing either system in isolation (Phakathi and Sinyolo 2025). While traditional forecasting and community‐level coping mechanisms remain invaluable, they must be augmented by technological innovations, including improved crop varieties, resilient irrigation infrastructure, and precision agriculture tools (Gamage et al. 2024). Furthermore, research‐driven innovation in crop genetics, climate modeling, and soil conservation is essential for bolstering long‐term resilience. Emerging digital technologies like artificial intelligence (AI) are playing an increasingly integral role in climate‐smart agriculture. AI‐enabled platforms, such as the *Plant Village Nuru* app, have demonstrated high diagnostic accuracy for in‐field diseases using smartphone imagery, thereby enhancing pest management. Similarly, digital mechanization platforms such as *Hello Tractor*, which utilize AI and the Internet of Things (IoT) to connect smallholders with tractor owners, and pioneered in Nigeria and Kenya respectively, have helped to optimize land preparation efficiency despite erratic rainfall patterns (Mrisho et al. 2020). Other sophisticated tools, such as the *Watson Decision Platform for Agriculture*, integrate climate, soil, and market data to provide AI‐driven insights for proactive disease management (Kaushik 2025). Collectively, these innovations, ranging from remote sensing to early warning systems, enable precision diagnostics and targeted interventions that mitigate the impacts of climatic stressors. However, this cannot be achieved without the provision of basic infrastructure, improved data accessibility, and farmer‐oriented capacity building. Accelerating adaptation efforts also requires the strengthening of policy and regulatory frameworks, particularly regarding climate governance (Masio 2023). Environmental stewardship, including forest conservation, reforestation, and soil health management, is equally critical, as these practices provide ecosystem services that buffer against climatic shocks. The study also highlights the impact of demographic shifts, specifically the migration of youth from rural areas to urban centers. Driven by inadequate infrastructure and limited rural opportunities, this trend significantly weakens the agricultural labor force. Incentivizing the youth through improved rural amenities, targeted financing, and agribusiness opportunities is vital for ensuring a sustainable agricultural economy in Africa.

**FIGURE 4:**
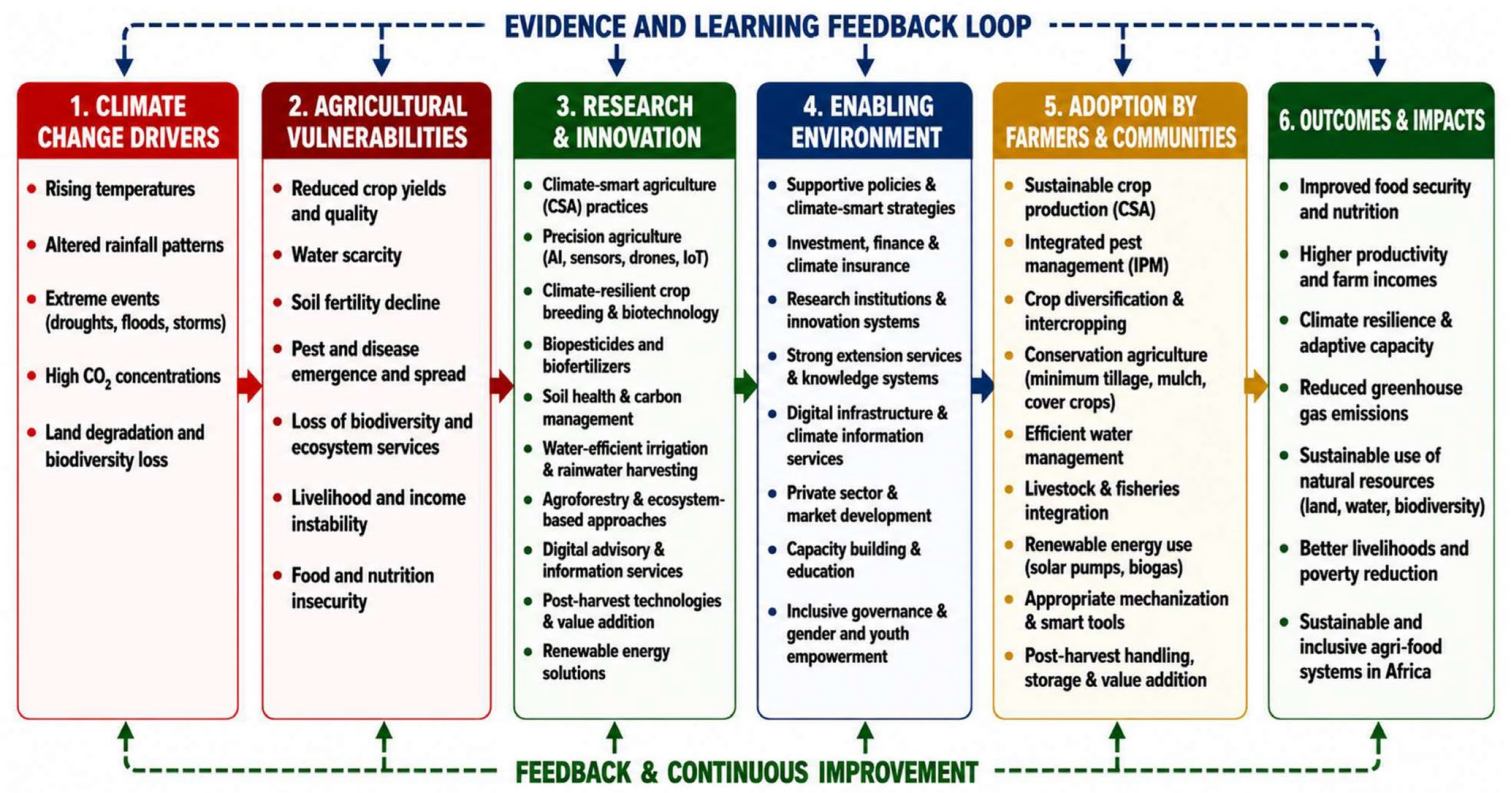
Framework for advancing food security and agriculture in Africa (Designed by authors using ChatGPT). This figure presents a conceptual framework illustrating the major climate change drivers and agricultural vulnerabilities facing Africa, together with key intervention pathways. These pathways include research and innovation, the establishment of an enabling environment, and the adoption of climate‐smart agricultural practices. Through continuous feedback, collaboration, and coordinated action among all stakeholders, these interventions have the potential to transform African agriculture, resulting in improved resilience, increased productivity, enhanced food security, and sustainable agricultural development.

Finally, this analysis emphasizes the need for enhanced coordination among academia, government, the private sector, and farming communities. Such collaboration is essential for translating scientific advancements into field‐level solutions. By fostering synergy among stakeholders and prioritizing innovation, environmental conservation, and inclusive governance, Africa can transform climate challenges into pathways for sustainable growth, food security, and long‐term agricultural transformation (Figure 4; Oniang'o et al. 2025).

### 4.1 Strategic Framework for Enhancing Agricultural Resilience in Africa

African agricultural resilience rests upon four foundational pillars: Systemic Synergy, Technological Innovation, Environmental Stewardship, and Inclusive Governance. To achieve these, the following pillars are essential.

#### 4.1.1 Integration of Indigenous Knowledge Systems (IKS)

A central finding is the necessity of an integrative framework that harmonizes indigenous knowledge systems (IKS) with modern technological interventions. As evidenced in South Africa, farmers who blend traditional forecasting and coping mechanisms with modern smallholder irrigation and precision tools achieve superior productivity and cost‐efficiency compared to those utilizing a single epistemological approach (Phakathi and Sinyolo 2025; Gamage et al. 2024). IKS encompasses a repository of longitudinal observations and coping mechanisms developed over generations to survive climatic extremes. Modern research should facilitate a synergy (Ijatuyi et al. 2025), where indigenous insights into local microclimates and traditional forecasting are integrated with scientific data to create robust, culturally relevant adaptation frameworks (Apraku et al. 2021). This epistemological integration will maximize adaptive capacity, mitigative structure, and ensure stronger resilience of rural communities in Africa.

#### 4.1.2 Enhanced Fiscal Instruments, and Sovereign Creditworthiness

Access to finance remains a fundamental prerequisite for adequate adaptation and building long‐term climate resilience. Strategic investment facilitates the dissemination of resilient cultivars, the construction of advanced irrigation infrastructure, the provision of storage facilities and efficient transport systems, and the funding of bio‐innovative research and precision technologies. Strategic investment in emerging agricultural technologies will be essential for improving the reliability, sustainability, and long‐term climate resilience of future food systems. Complementary shifts toward novel food sources and healthier dietary patterns can further reduce dependence on land, water, and chemical inputs, thereby minimizing the environmental impacts of climate change on agricultural production. Policies and investments that accelerate the adoption of validated innovations while fostering the development, commercialization, and large‐scale implementation of next‐generation technologies, sustainable production systems, and alternative food products should be prioritized to advance resilient and sustainable global food systems (Yang et al. 2024). However, many African nations face significant barriers to climate financing due to high debt‐to‐GDP ratios and governance deficits that compromise sovereign creditworthiness. To overcome these constraints, leadership must prioritize fiscal transparency and the implementation of selfless, evidence‐based policy reforms. Also, strengthening regulatory frameworks related to carbon markets, land tenure, and climate governance is essential for accelerating the pace of adaptation, mitigation and long‐term agricultural resilience to harsh climate (Masio 2023).

#### 4.1.3 Demographic Revitalization and Youth Incentivization

This study underscores a troubling demographic shift: the accelerating migration of youth from rural agricultural hubs to urban centers, driven by a lack of infrastructure and perceived economic stagnation. This rural‐to‐urban flight depletes the agricultural labor force and threatens the long‐term sustainability of the agricultural sector. Incentivizing youth participation through the agribusiness model supported by digital tools, targeted financing, and improved rural amenities is vital for revitalizing rural economies and mitigating the socio‐economic challenges of youth unemployment. Governments must prioritize investment in telecommunications, energy, and transport, especially in rural communities. Furthermore, providing concessional financing (soft loans) and agribusiness incentives can encourage and empower youth entrepreneurs to participate effectively in reviving the sector.

#### 4.1.4 Preservation of Soil Health and Integrity

Sustaining agricultural productivity requires the maintenance of soil microbial health and structural integrity. Conservative agricultural practices such as the application of organic amendments, biopesticides, and efficient hydrological management are essential to ensure that agricultural soils can withstand unpredictable climatic shocks. Deliberate soil conservation efforts must focus on maintaining moisture retention and nutrient profiles to buffer against the aridification associated with global warming (Brempong et al. 2023). Chemical applications that pose environmental hazards should be avoided or reduced as much as possible. Enhancing our understanding of microbial contributions to soil health and ecosystem resilience under climate change will provide valuable insights for farmers, land managers, and ecosystem restoration practitioners in designing and implementing climate‐smart agricultural and ecological systems (Raza et al. 2025).

#### 4.1.5 Transdisciplinary Synergy and Knowledge Dissemination

The successful implementation of climate‐smart policies requires a united effort across academia, the private sector, government bodies, and farming communities. Currently, the siloing of research outputs often prevents the transfer of scientific knowledge to field‐level practitioners. Establishing collaborative platforms for knowledge exchange ensures that academic innovations are translated into actionable, farm‐level solutions. Such synergy fosters a cohesive response to climate stressors, reducing institutional fragmentation and accelerating the adoption of novel technologies (Brempong et al. 2023). The government must intentionally foster harmony among various agricultural stakeholders by creating regular interactive avenues via conferences, symposiums, seminars, and webinars where ideas and strategies can be harmonized.

#### 4.1.6 Deployment of Artificial Intelligence (AI) and Machine Learning

Prioritization of innovation such as the deployment of artificial intelligence (AI) has emerged as a transformative force in modern crop improvement, enabling researchers and breeders to address the growing demand for sustainable and climate‐resilient agricultural systems. By integrating large‐scale genomic, phenotypic, and environmental datasets with advanced predictive models, AI enables breeders to move beyond the limitations of conventional breeding approaches toward more precise, efficient, and data‐driven crop improvement strategies (Feng et al. 2024). AI‐assisted prediction of agronomically important traits before planting can substantially shorten breeding cycles, reducing processes that traditionally require several years to a single growing season, thereby accelerating the development of superior crop varieties (Feng et al. 2024).

AI‐driven platforms, such as the Nuru app (Plant Village), facilitate real‐time, high‐accuracy in‐field disease diagnostics via smartphone imagery, significantly improving pest management outcomes for smallholders. Similarly, “Hello Tractor” utilizes “internet of things” (IoT) to optimize tractor‐hiring services, reducing transaction costs and improving land preparation efficiency (Mrisho et al. 2020). Furthermore, the Watson Decision Platform demonstrates the potential of integrating soil, climate, and market data into actionable, AI‐driven insights (Kaushik 2025). AI‐enabled models can synthesize satellite imagery and longitudinal meteorological data to enhance the precision of early warning systems for both weather anomalies and pest outbreaks (Kaushik 2025). Furthermore, AI‐powered platforms can deliver localized recommendations in indigenous languages, advising farmers on optimal planting windows and resilient cultivar selection (Omotayo et al. 2025).

The integration of AI into crop breeding has also transformed traditional decision‐making frameworks, shifting breeding practices from experience‐based approaches to intelligent, data‐driven systems capable of optimizing selection accuracy and breeding efficiency (Fu et al. 2025). Coupled with high‐speed breeding robotics, AI‐generated breeding decisions can be executed with exceptional precision through automated operations such as seed sowing, phenotypic imaging, tissue sampling, and data collection under both greenhouse and field conditions. Equipped with high‐throughput imaging technologies and advanced sensor platforms, these robotic systems continuously monitor plant growth, environmental conditions, and molecular responses, generating real‐time datasets that further enhance AI‐driven breeding models and facilitate the rapid development of climate‐smart crop varieties (Fu et al. 2025). Government must however provide basic infrastructure that can make technological advancement possible by providing improved data accessibility and adequate capacity building for farmers, especially smallholders.

#### 4.1.7 Advancement in the Application of Genomics and More Complex Digital Technologies

**FIGURE 5:**
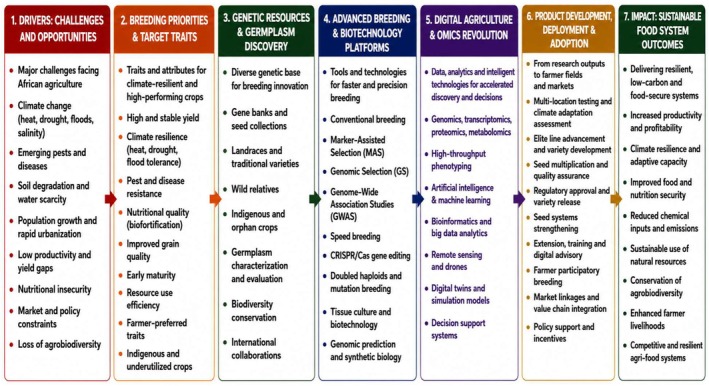
Pathway of advancement from challenges to impact. (Designed by authors using ChatGPT). This figure shows a framework for Next‐Generation plant breeding and agricultural innovation to advance Food Security and Sustainable Agriculture in Africa. This can be achieved through science‐led innovation, strong partnerships and collaborations, adoption and implementation of climate smart strategies, and inclusive approaches.

Advanced biotechnological tools, such as the CRISPR/Cas‐based gene editing and transgenic breeding, allow scientists to manipulate plant genomes by introducing or transferring genes with desirable attributes, like resistance to diseases, pests, or abiotic stresses, giving rise to smarter crops (Raza et al. 2025). Smart crops are engineered to possess superior adaptive capacity across a wide range of environmental conditions, enabling them to maintain growth, productivity, and resilience under increasingly variable and unpredictable climates. By effectively balancing physiological trade‐offs associated with stress adaptation, these crops can tolerate multiple biotic and abiotic stresses without substantial reductions in growth or yield. Furthermore, smart crops incorporate traits that enhance resource‐use efficiency, maximize net productivity, and minimize environmental impacts, making them a cornerstone of climate‐smart and sustainable agricultural systems aimed at ensuring long‐term food security (Wang et al. 2024). This precision bred crops help to effectively address malnutrition, food insecurity and promote sustainable agriculture. The era of single and integrated plant omics has helped to discover numerous plant genes and created a better understanding of stress adaptation and tolerance in plants (Raza et al. 2025). For further advancement and progress in plant technology and modification, cutting‐edge technologies, such as; genome sequencing, high‐throughput phenotyping platforms, precise genome‐editing tools, and synthetic biology methods among others must be adequately employed, (Wang et al. 2024) to enable a smooth transition from vulnerability to impact in the African agricultural system (Figure 5).

#### 4.1.8 Inclusion of Climate Governance Into Country Policies by African Governments

A critical deficit in African climate management is the absence of specific regulatory guidelines governing the intersection of climatic activity and agriculture. Many African nations currently lack standardized climate policies, creating a governance vacuum where environmental management is often left to chance rather than strategic planning. This lack of oversight has significantly exacerbated the vulnerabilities of African agriculturists. To mitigate these impacts, governments must proactively integrate climate governance into national policy frameworks. Specifically, policies should be designed to institutionalize environmental stewardship and address the demographic drivers of agricultural decline. This includes incentivizing rural residency through strategic infrastructural investment, thereby curbing the accelerated migration of youth to urban centers and preserving the labor force essential for the continent's arable lands.

#### 4.1.9 Fiscal Monitoring and Evaluation Frameworks

Financial investment in African climate management must be matched by a commitment to fiscal integrity. To combat the systemic challenges of corruption and fund diversion, African governments should implement stringent oversight on all climate‐related expenditures. Resources must be strictly dedicated to their stipulated agricultural or environmental purposes, backed by a comprehensive auditing process. Establishing independent monitoring and evaluation (M&E) frameworks will ensure that appropriation aligns with national resilience goals. Accountability must be strictly enforced; those found guilty of financial mismanagement should face legal consequences to discourage future malpractice. A transparent, multi‐stakeholder approach to climate finance is non negotiable for the successful implementation of long‐term strategies.

## 5 Conclusions and Future Perspectives

Unpredictable climatic variations, continue to disrupt African agricultural systems, intensifying the risks of poverty and food insecurity. In response, the continent is increasingly adopting a diverse range of adaptive strategies, from improved crop cultivars and water‐smart infrastructure to advanced digital advisory platforms. However, more effort is required in stakeholder synergy, efficient use of digital technologies, smart agricultural practices, adequate integration of indigenous knowledge and integration of climate governance into the central policies of African countries. The convergence of nano biotechnology, gene editing, speed breeding, microbial engineering, and the exploitation of beneficial plant–microbe interactions, combined with a comprehensive understanding of plant stress physiology, represents a transformative strategy for developing future stress‐smart crops capable of sustaining agricultural productivity under increasingly unstable climatic conditions. These complementary approaches have the potential to accelerate the development of resilient crop varieties with enhanced tolerance to multiple biotic and abiotic stresses, thereby contributing significantly to sustainable agriculture and global food security.

Furthermore, emerging technologies such as pan‐genomics, synthetic genomics, high‐throughput phenotyping, remote sensing, and artificial intelligence (AI) are redefining modern crop improvement by enabling faster, more precise, and data‐driven breeding strategies. The integration of these technologies, particularly AI‐assisted genomic prediction and high‐throughput phenotyping, can substantially accelerate the identification, selection, and deployment of climate‐smart crop varieties (Raza et al. 2025). Looking ahead, the convergence of next‐generation AI, functional genomics, and heterogeneous big‐data platforms will transform breeding technologies into hyperintelligent systems capable of atomic‐level precision in trait discovery and manipulation. Such innovations will provide robust technological solutions for addressing pressing global challenges, including food security, climate change adaptation, sustainable resource utilization, and resilient agricultural production systems (Fu et al. 2025).

Artificial intelligence (AI) continuously refines deep learning models to identify disease‐resistance traits, predict the effects of environmental variability on crop performance, and simulate optimal genetic combinations for breeding. These capabilities accelerate climate adaptation and enable targeted crop improvement, providing sustainable solutions for enhancing agricultural productivity and global food security (Fu et al. 2025). By integrating diverse datasets, AI establishes a comprehensive multimodal framework for understanding crop growth and development. This holistic approach enhances the precision of breeding decisions by enabling accurate prediction of complex trait performance across diverse environments. Furthermore, the integration of AI with robotic breeding platforms transforms conventional crop improvement into a highly automated, computational, and data‐driven process, increasing breeding efficiency and positioning AI‐enabled breeding systems as a cornerstone of future climate‐resilient agriculture and global food security (Fu et al. 2025).

Young people must also be encouraged to participate in agrobusiness by building infrastructural support especially in the rural areas where a major portion of the arable land is located, this will help to curtail youthful exit from the rural areas that reduces the agricultural workforce. Extension services must be made available to farmers to acquaint them with recent technologies that can help them to build stronger resilience to climate change impact. It is essential to develop monitoring and evaluation frameworks that ensure that allocated funds are translated into tangible on‐the‐ground outcomes. Empirical attention should be directed toward identifying risks of fund misallocation and the structural political factors that undermine fiscal transparency. A knowledge gap still exists in climatic weather conditions in some African countries including Somalia and North Africa. Further research should assess the vulnerabilities of these countries to climate change and also analyze the adaptation strategies, if any. Future research should also prioritize field‐based empirical studies to obtain primary data regarding farmers' experiences. Direct engagement with agricultural practitioners and stakeholders is essential to derive conclusions grounded in practical realities.

### 5.1 Limitations

While this review provides a broad synthesis of the 2015–2025 decade, it acknowledges certain limitations inherent to the comprehensive review format. First, even though a structured search strategy was employed across credible databases, the selection of literature may be subject to inherent selection bias, as the study prioritizes peer‐reviewed evidence that aligns with the regional comparative framework. Secondly, the review is qualitative in nature, synthesizing diverse datasets that often utilize different metrics for “resilience” and “productivity” which may limit direct cross‐national statistical comparability.

## Funding

This work was supported by the International Centre for Genetic Engineering and Biotechnology, ZAF/DSI/WESTAR/10.

## Ethics Statement

The authors have nothing to report.

## Conflicts of Interest

The authors declare no conflicts of interest.

## Data Availability

Data sharing is not applicable to this article as no new data were created or analyzed during this study. All sources analyzed are included in the reference list of this published article.
